# Retraction notice to “Influence of nanotechnology to combat against COVID-19 for global health emergency: A review” [Sens. Int. 2 (2021) 100079]

**DOI:** 10.1016/j.sintl.2026.100383

**Published:** 2026-05-01

**Authors:** Aswini Rangayasami, Karthik Kannan, S. Murugesan, Devi Radhika, Kishor Kumar Sadasivuni, Kakarla Raghava Reddy, Anjanapura V. Raghu

**Affiliations:** a Department of Botany, Periyar University, Salem, 636 011, India; b Center for Advanced Materials, Qatar University, P.O Box 2713, Doha, Qatar; c Department of Chemistry, Faculty of Engineering and Technology, Jain Deemed-to-be University, Ramnagara, 562112, Karnataka, India; d School of Chemical and Biomolecular Engineering, The University of Sydney, Sydney, NSW, 2006, Australia

This article has been retracted: please see Elsevier policy on article withdrawal (https://www.elsevier.com/about/policies-and-standards/article-withdrawal).

This article has been retracted at the request of the Editor.

Post-publication, concerns were raised on PubPeer that the article contains “tortured phrases” that are not understandable, meaningful text. The journal editor assessed the articles and found more phrases that deviate from standard technical terms. The authors were requested to explain the use of these passages of text but were unable to do so.

Additionally, an investigation conducted on behalf of the journal by Elsevier's Research Integrity & Publishing Ethics team has determined that the authors were requested by one of the editors and one of the reviewers to insert redundant references to their paper during the peer-review process.

The investigation also found that review of this submission was handled by the Editor-in-Chief (Tejraj Aminabhavi) despite an extensive record of collaboration, including co-publication, with one of the paper authors (Anjanapura V. Raghu). This compromised the editorial process and breached the journal's policies.

Further to the above concerns, the investigation identified a change in authorship between the original submission and the revised version of this paper. During revision, the author (Kakarla Raghava Reddy) was added to the revised paper without explanation and without exceptional approval by the journal editor, which is contrary to the journal policy on changes to authorship.

The Editor has lost confidence in the findings of the article and has determined that it should be retracted.

The authors disagree with the retraction and dispute the grounds for it.

